# Associations between chlorophyll *a *and various microcystin health advisory concentrations

**DOI:** 10.12688/f1000research.7955.2

**Published:** 2016-06-13

**Authors:** Jeffrey W. Hollister, Betty J. Kreakie

**Affiliations:** 1 Office of Research and Development, National Health and Environmental Effects Research Laboratory, Atlantic Ecology Division, US Environmental Protection Agency, Narragansett, RI, USA

**Keywords:** Harmful Algal Blooms, Cyanotoxins, National Lakes Assessment, Conditional Probability Analysis, Cyanobacteria

## Abstract

Cyanobacteria harmful algal blooms (cHABs) are associated with a wide range of adverse health effects that stem mostly from the presence of cyanotoxins. To help protect against these impacts, several health advisory levels have been set for some toxins. In particular, one of the more common toxins, microcystin, has several advisory levels set for drinking water and recreational use. However, compared to other water quality measures, field measurements of microcystin are not commonly available due to cost and advanced understanding required to interpret results. Addressing these issues will take time and resources. Thus, there is utility in finding indicators of microcystin that are already widely available, can be estimated quickly and *in situ*, and used as a first defense against high levels of microcystin. Chlorophyll *a* is commonly measured, can be estimated *in situ*, and has been shown to be positively associated with microcystin. In this paper, we use this association to provide estimates of chlorophyll *a* concentrations that are indicative of a higher probability of exceeding select health advisory concentrations for microcystin. Using the 2007 National Lakes Assessment and a conditional probability approach, we identify chlorophyll *a *concentrations that are more likely than not to be associated with an exceedance of a microcystin health advisory level. We look at the recent US EPA health advisories for drinking water as well as the World Health Organization levels for drinking water and recreational use and identify a range of chlorophyll *a* thresholds. A 50% chance of exceeding one of the specific advisory microcystin concentrations of 0.3, 1, 1.6, and 2 μg/L is associated with chlorophyll *a* concentration thresholds of 23, 68, 84, and 104 μg/L, respectively. When managing for these various microcystin levels, exceeding these reported chlorophyll *a* concentrations should be a trigger for further testing and possible management action.

## Introduction

Over the last decade, numerous events and legislative activities have raised the public awareness of harmful algal blooms ^1– 3^. In response the US Environmental Protection Agency (USEPA) has recently released suggested microcystin (one of the more common toxins) concentrations that would trigger health advisories ^4– 6^. Additionally, the World Health Organization (WHO) has microcystin advisory levels for drinking water and for a range of recreational risk levels ^7, 8^. While these levels and associated advisories are likely to help mitigate the impacts from harmful algal blooms, they are not without complications.

One of these complications is that they rely on available measurements of microcystin. While laboratory testing (e.g., chromatography) remains the gold standard for quantifying microcystin concentrations in water samples, several field test kits have been developed. Even though field tests provide a much needed means for rapid assessment, they are not yet widely used and are moderately expensive (approximately $150–$200 depending on specific kit) with a limited shelf life (typically one year) ^9, 10^. Additionally, each technique requires nuanced understanding of the detection method (e.g., limit of detection, specific microcystin variants being measured, and sampling protocol).

Fortunately, cyanobacteria and microcystin-LR has been shown to be associated with several other, more commonly measured and well understood components of water quality that are readily assessed in the field ^11^. For instance, there are small or hand held fluorometers that measure chlorohpyll *a*. Additionally, chlorophyll *a* is a very commonly measured component of water quality that is also known to be positively associated with microsystin-LR concentrations ^12, 13^. Recently, Yuan *et al.* ^13^ explored these associations in detail and controlled for other related variables. In their analysis they find that total nitrogen and chlorophyll *a* show the strongest association with microcystin. Furthermore, they identify chlorophyll *a* and total nitrogen concentrations that are associated with exceeding 1 *µ*g/L of microcystin. These findings suggest that chlorophyll *a* concentrations could also track the new USEPA microcystin health advisory levels for drinking water. Identifying this association would provide an important tool for water resource managers to help manage the threat to public health posed by cHABs and would be especially useful in the absence of measured microcystin concentrations.

In fact, this is a similar tact to the World Health Organization who, in addition to advisory levels for microcystin, have also proposed related advisory levels for cyanobacteria abundance and chlorophyll *a* ^7, 8^. The chlorophyll *a* concentrations proposed by the WHO are for low (< 10 *µ*g/L), moderate (between 10 and 50 *µ*g/L, high (between 50 and 5000 *µ*g/L), and very high risk (>5000 *µ*g/L) ^8^. While these advisories have proven to be useful tools they do suffer from being coarse, broad, and have been found to overestimate actual risk ^14^.

In this paper we build on these past efforts and utilize the National Lakes Assessment (NLA) data and identify chlorophyll *a* concentrations that are associated with higher probabilities of exceeding several microcystin health advisory concentrations ^6, 8, 15^. We build on past studies by exploring associations with the newly announced advisory levels and by also applying a different method, conditional probability analysis. Utilizing different methods strengthens the evidence for suggested chlorophyll *a* levels that are associated with increased risk of exceeding the health advisory levels as those levels are not predicated on a single analytical method. So that others may repeat or adjust this analysis, the data, code, and this manuscript are freely available via https://github.com/USEPA/microcystinchla.

## Methods

### Data

We used the 2007 NLA chlorophyll *a* and microcystin-LR concentration data ^15^. These data represent a snapshot of water quality from the summer of 2007 for the conterminous United States and were collected as part of an ongoing probabilistic monitoring program ^15^. Water quality data, including chlorophyll *a* and microcystin-LR were obtained via an integrated sample taken from the surface of the lake down to 2 meters. Samples were taken at the same time from the index site (e.g. near the centroid of the lake) and these provide the source for both chlorophyll *a* and microcystin-LR ^15^.

For our analysis we only used samples that were part of the probability sampling design (i.e. no reference samples) and from the first visit to the lake (e.g. some lakes were sampled multiple times). The detection limit for microcystin-LR was 0.05 *µ*g/L. Approximately 67% of lakes reported microcystin-LR at the detection limit. For this analysis we retained these values as removing them would erroneously reduce the confidence intervals around the conditional probabilities. Data on chlorophyll *a* and microcystin-LR concentrations are available for 1028 lakes.

### Analytical methods

We used a conditional probability analysis (CPA) approach to explore associations between chlorophyll *a* concentrations and World Health Organization (WHO) and USEPA microcystin health advisory levels ^17^. Many health advisory levels have been suggested ( Table 1), but lakes with higher microcystin-LR concentrations in the NLA were rare. Only 1.16% of lakes sampled had a concentration greater than 10 *µ*g/L. Thus, for this analysis we focused on the microcystin concentrations that are better represented in the NLA data. These were the USEPA children’s (i.e. bottle fed infants to pre-school age children) drinking water advisory level of 0.3 *µ*g/L (USEPA Child), the WHO drinking water advisory level of 1 *µ*g/L (WHO Drinking), the USEPA adult (i.e. beyond pre-school aged individuals) drinking water advisory level of 1.6 *µ*g/L (USEPA Adult), and the WHO recreational, low probability of effect advisory level of 2 *µ*g/L (WHO Recreational).

**Table 1.** Various microcystin health advisory concentrations from the USEPA and World Health Organization.

Source	Type	Concentration
USEPA	Child Drinking Water Advisory	0.3 *µ*g/L
WHO	Drinking Water	1 *µ*g/L
USEPA	Adult Drinking Water Advisory	1.6 *µ*g/L
WHO	Recreational: Low Prob. of Effect	2–4 *µ*g/L
WHO	Recreational: Moderate Prob. of Effect	10–20 *µ*g/L
WHO	Recreational: High Prob. of Effect	20–2000 *µ*g/L
WHO	Recreational: Very High Prob. of Effect	>2000 *µ*g/L

Conditional probability analysis provides information about the probability of observing one event given another event has also occurred. For this analysis, we used CPA to examine how the conditional probability of exceeding one of the health advisories changes as chlorophyll *a* increases in a lake. We expect to find higher chlorohpyll *a* concentrations to be associated with higher probabilities of exceeding the microcystin health advisory levels. We also calculated bootstrapped 95% confidence intervals (CI) using 10,000 bootstrapped samples. Thus, to identify chlorophyll *a* concentrations of concern we identified the value of the upper 95% CI across a range of conditional probabilities of exceeding each health advisory level. Using the upper confidence limit to identify a threshold is justified as it ensures that a given threshold is unlikely to miss a microcystin exceedance.

As both microcystin-LR and chlorophyll *a* values were highly right skewed, a log base 10 transformation was used. Additional details of the specific implementation are available at https://github.com/USEPA/microcystinchla. A more detailed discussion of CPA is beyond the scope of this paper, but see Paul *et al.* ^18^ and Hollister *et al.* ^19^ for greater detail. All analyses were conducted using R version 3.2.3 and code and data from this analysis are freely available as an R package at https://github.com/USEPA/microcystinchla.

Lastly, we assessed the ability of these chlorophyll *a* thresholds to predict microcystin exceedance. We used error matrices and calculate total accuracy as well as the proportion of false negatives. Total accuracy is the total number of correct predictions divided by total observations. The proportion of false negatives is the total number of lakes that were predicted to not exceed the microcystin guidelines but actually did, divided by the total number of observations.

## Results

**Figure 1.**
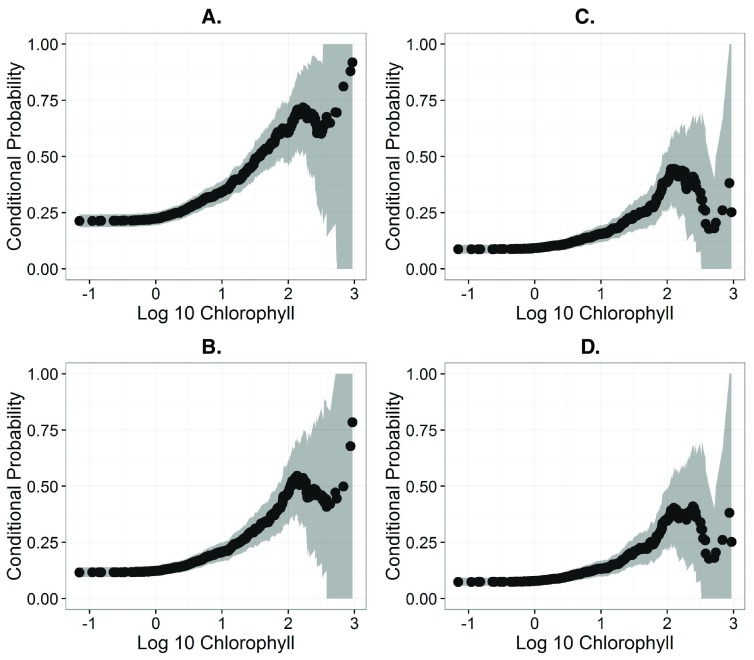
Conditional probability plots showing association between the probability of exceeding various microcystin-LR (MLR) health advisory Levels. **A**.) Plot for USEPA Child (0.3 *µ* g/L). **B**.) Plot for WHO Drinking (1 *µ *g/L). **C**.) Plot for USEPA Adult (1.6 *µ *g/L). **D**.) Plot for WHO Recreational (2 *µ *g/L).

In the 2007 NLA, microcystin-LR concentrations ranged from 0.05 to 225 *µ*g/L. Microcystin-LR concentrations of 0.05 *µ*g/L represent the detection limits. Any value greater than that indicates the presence of microcystin-LR. Of those lakes with microcystin-LR, the median concentration was 0.51 *µ*g/L and the mean was 3.17 *µ*g/L. Of all lakes sampled, 21% of lakes exceeded the USEPA Child level, 8.8% of lakes exceeded the USEPA Adult level, 11.7% of lakes exceeded the WHO Drinking level, and 7.3% of lakes exceeded the WHO Recreational level. Chlorophyll *a*, ranged from 0.07 to 936 *µ*g/L and this captures the range of trophic states from oligotrophic to hypereutrophic. All lakes had detectable levels of chlorophyll *a*. The median concentration was 7.79 *µ*g/L and the mean was 29.63 *µ*g/L. The association between chlorophyll *a* and the upper confidence interval across a range of conditional probability values are shown in Table 2. Specific chlorophyll *a* concentrations that are associated with greater than even odds of exceeding the advisory levels were 23, 68, 84, and 104 *µ*g/L for 0.3, 1.0, 1.6, and 2.0 *µ*g/L advisory levels, respectively ( Table 2 & Figure 1).

The chlorophyll *a* cutoffs may be used to predict whether or not a lake exceeds the microcystin health advisories. Doing so allows us to compare the accuracy of the prediction as well as evaluate false negatives. Total accuracy of the four cutoffs predicting microcystin exceedances were 74% for the USEPA children’s drinking water advisory, 86% for the WHO drinking water advisory, 89% for the USEPA adult drinking water advisory, and 91% for the WHO recreational advisory ( Table 3, Table 4, Table 5, & Table 6). However, total accuracy is only one part of the prediction performance with which we are concerned.

**Table 2.** Chlorophyll *a* concentrations that are associated with a 50% probability of exceeding a microcystin health advisory concentration.

Cond. Probability	USEPA Child (0.3 *µ*g/L)	WHO Drink (1 *µ*g/L)	USEPA Adult (1.6 *µ*g/L)	WHO Recreational (2 *µ*g/L)
0.1	0.07	0.07	0.07	1
0.2	0.07	4	12	17
0.3	3	17	32	45
0.4	11	37	68	77
0.5	23	68	84	104
0.6	39	97	115	185
0.7	66	126	871	871
0.8	116	271	871	871
0.9	170	516	871	871

**Table 3.** Confusion matrix comparing chlorophyll *a* predicted exceedences (rows) versus real exceedances (columns) for the USEPA childrens drinking water advisory.

	Not Exceed	Exceed	Row Totals
Not Exceed	643	95	738
Exceed	168	122	290
Column Totals	811	217	1028

**Table 4.** Confusion matrix comparing chlorophyll *a* predicted exceedences (rows) versus real exceedances (columns) for the WHO drinking water advisory.

	Not Exceed	Exceed	Row Totals
Not Exceed	841	78	919
Exceed	66	43	109
Column Totals	907	121	1028

**Table 5.** Confusion matrix comparing chlorophyll *a* predicted exceedences (rows) versus real exceedances (columns) for the USEPA adult drinking water advisory.

	Not Exceed	Exceed	Row Totals
Not Exceed	884	57	941
Exceed	53	34	87
Column Totals	937	91	1028

**Table 6.** Confusion matrix comparing chlorophyll *a* predicted exceedences (rows) versus real exceedances (columns) for the WHO recreational water advisory.

	Not Exceed	Exceed	Row Totals
Not Exceed	908	51	959
Exceed	45	24	69
Column Totals	953	75	1028

When using the chlorophyll *a* cutoffs as an indicator of microcystin exceedances, the error that should be avoided is predicting that no exceedance has occurred when in fact it has. In other words, we would like to avoid Type II errors and minimize the proportion of false negatives. For the four chlorophyll *a* cut-offs we had a proportion of false negatives of 9%, 8%, 6%, and 5% for the USEPA children’s, the WHO drinking water, the USEPA adult, and the WHO recreational advisories, respectively. In each case we missed less than 10% of the lakes that in fact exceeded the microcystin advisory. While this method performs well with regard to the false negative percentage, it is possible that is a relic of the NLA dataset and testing with additional data would allow us to confirm this result.

## Discussion

**Figure 2.**
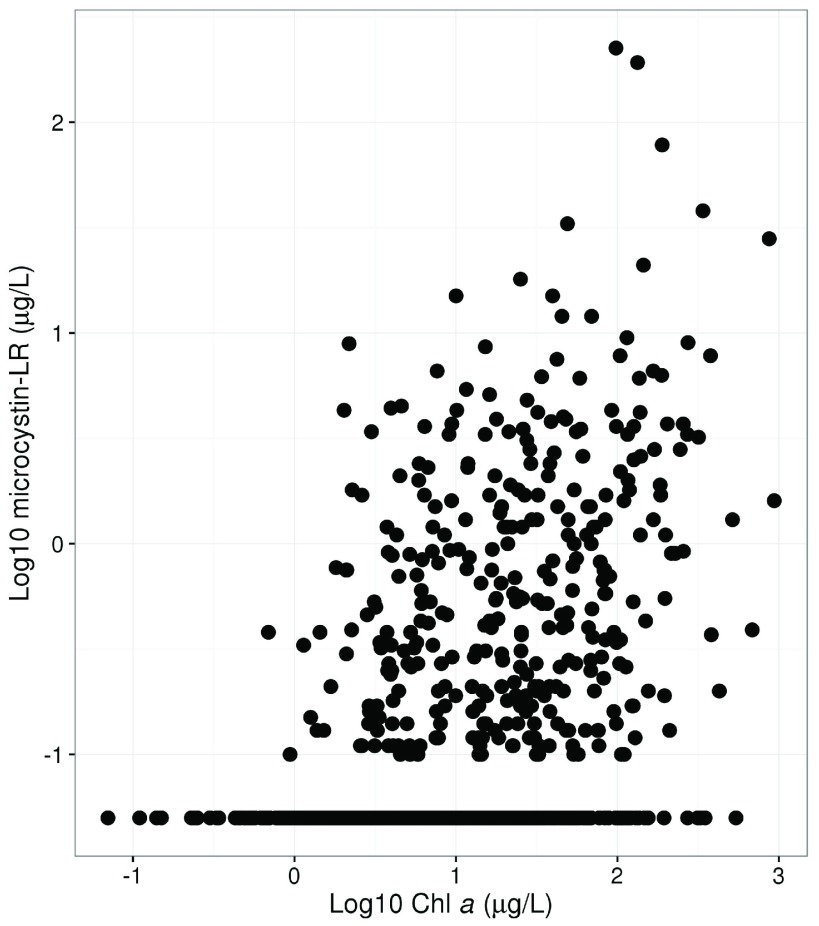
Scatterplot showing association between chlorophyll *a* and microcystin-LR.

The log-log association between microcystin-LR and chlorophyll *a* indicates that, in general, higher concentrations of microcystin-LR almost always co-occur with higher concentrations of chlorophyll *a* yet the inverse is not true ( Figure 2). Higher chlorophyll *a* is not necessarily predictive of higher microcystin-LR concentrations; however, chlorophyll *a* may be predictive of the probability of exceeding a certain threshold.

Indeed, the probability of exceeding each of the four tested health advisory levels increased as a function of chlorophyll *a* concentration ( Figure 1). We used this association to identify chlorophyll *a* concentrations that were associated with a range of probabilities of exceeding a given health advisory level ( Table 2). For the purposes of this discussion we focus on a conditional probability of 50% or greater (i.e., greater than even odds to exceed a health advisory level). The 50% conditional probability chlorophyll *a* thresholds represents 28.6%, 11%, 8.9%, and 7.2% of sample lakes for the USEPA Child, the WHO Drinking, the USEPA Adult, and the WHO recreational levels, respectively.

There are numerous possible uses for the chlorophyll *a* and microcystin advisory cut-off values. First, in the absence of microcystin-LR measurements, exceedence of the chlorophyll *a* concentrations could be a trigger for further actions. Given that there is uncertainity around these chlorophyll *a* cutoffs the best case scenario would be to monitor for chlorophyll *a* and in the event of exceeding a target concentration take water samples and have those samples tested for microcystin-LR.

A second potential use is to identify past bloom events from historical data. As harmful algal blooms are made up of many species and have various mechanisms responsible for adverse impacts (e.g., toxins, hypoxia, odors), there is no single definition of a bloom. For cHABs, one approach has been to utilize phycocyanin to screen for or identify bloom events ^20^. This is a useful approach, but phycocyanin is not always available, thus limiting its utility especially for examining historical data. Using our chlorophyll *a* cutoffs provides a value that is also associated with microcystin-LR and can be used to classify lakes, from past surveys, as having bloomed.

The values we propose are national and may miss regional variation in water quality, including, chlorophyll *a* and microcystin-LR ^22^. A set of regional conditional probabilities would be interesting; however, limiting the analysis to the data available per region would make interpretation difficult. The sample size for each of the regional conditional probabilities would be reduced and the number of lakes in each region that exceed the microcystin values would also be reduced. Thus, our confidence in the conditional probabilities would be less (i.e. greatly increased confidence intervals) and the relationships less pronounced as we have fewer lakes on which to base the probabilities. Thus, this dataset is best for making national scale recommendations.

There are two other limitations with the 2007 National Lakes Assessment dataset. First, it represents a single sample from a lake and does not capture temporal dynamics. Second, validation of the predictions with the 2007 data alone would be challenging as the data would need to be subset and this would only sever to increase the uncertainty of our conditional probabilities, reducing our ability to validate the presence of microcystin-LR. The 2012 National Lakes Assessment would be ideal for this task. However, as of this writing, the 2012 National Lakes Assessment data are not public. When these data are released, a validation of this approach can be completed then.

Lastly, using chlorophyll *a* is not meant as a replacement for testing of microcystin-LR or other toxins. It should be used when other, direct measurements of cyanotoxins are not available. In those cases, which are likely to be common at least in the near future, using a more ubiquitous measurement such as chlorophyll *a* will provide a reasonable proxy for the probability of exceeding a microcystin health advisory level and provide better protection against adverse effects in both drinking and recreational use cases.

## Data and software availability

### Data and latest source code

https://github.com/USEPA/microcystinchla

### Archived data and source code at time of publication

http://dx.doi.org/10.5281/zenodo.55273 ^24^

### License

Creative Commons Zero 1.0: http://creativecommons.org/publicdomain/zero/1.0/
