# Supplementary material for: Associations between chlorophyll a and various microcystin health advisory concentrations
Source: F1000Res. 2016 Jun 13;5:151. Originally published 2016 Feb 9. [Version 2] doi: 10.12688/f1000research.7955.2 (PMC4830210; doi:10.12688/f1000research.7955.2)
Supplement: Peer review history

# Peer review: Referee response for version 2

**DOI:** 10.5256/f1000research.9675.r14343

**Published:** 2016-06-28

**Authors:** Zofia E Taranu

**Affiliations:** 1 Department of Biological Sciences, Université de Montréal, Montreal, QC, Canada

I have read this submission. I believe that I have an appropriate level of expertise to confirm that it is of an acceptable scientific standard.

# Peer review: Referee response for version 1

**DOI:** 10.5256/f1000research.8562.r12719

**Published:** 2016-04-11

**Authors:** Zofia E Taranu

**Affiliations:** 1 Department of Biological Sciences, Université de Montréal, Montreal, QC, Canada

**General comments**

This study provides an elegant framework to predict the severe impairment of U.S. lakes and reservoirs by cyanobacterial harmful algal blooms. I especially appreciated the clever use of conditional probability analysis to identify chlorophyll *a* threshold above which MC concentrations exceed WHO drinking water and recreational provisional guidelines. Chlorophyll *a* is a regularly measured water quality variable, and this study indeed offers a promising approach that can me used in many lakes around the world to identify problematic lakes or regions. In that regard, it would have been interesting to account for the spatial heterogeneity across this landscape -- as indicated by Beaver *et al.* (2014), some regions of the continental U.S. are more likely to be MC hotspots. Accounting for this heterogeneity will likely help explain some of the noise in the biplot shown in Fig. 2. I have also analyzed the MC data from the same dataset and found that accounting for different ecoregions in my model (as presented in Beaver *et al.,* 2014) helped further explain the probability of detecting versus failing to detect MC in lakes. I am curious to know how this would play out with your modeling approach (conditional probability analysis).

**Minor comments**

I recommend the following minor typographical corrections:

**P3**

Change: “Yuan *et al.* (2014) explore these associations in detail and control for other related variables. In their analysis they find that total [...]”

To: “Recently, Yuan *et al.* (2014) explored these associations in detail and controlled for other related variables and found that total [...]”

Change: “Given these facts, it should be possible to identify chlorophyll a concentrations that would be associated with the [...]”

To:  “These findings suggest that chlorophyll a concentrations could also track the [...]”

Change: “Identifying these associations would provide another tool for [...]”

To: “Identifying this association would provide an important tool for [...]”

Change: “We add to past studies by exploring associations with newly announced advisory [...]”

To: “We build on past studies by exploring associations with the newly announced advisory [...]”

**P4**

Change: “Thus, to identify chlorophyll a concentrations of concern we identify the value [...]”

To: “Thus, to identify chlorophyll a concentrations of concern we identified the value [...]”

Change: “were highly skewed right,”

To: “were highly right skewed,”

Change: “Lastly, we assess the ability of”

To: “Lastly, we assessed the ability of”

Change: “We use error matrices and calculate total accuracy”

To: “We used error matrices and calculate total accuracy”

Change: “For chlorophyll a, the range was”

To: “Chlorophyll a ranged from”

Please specify that this chlorophyll a range corresponds to a range from oligotrophic to hypereutrophic lakes.

Change: “The associations between chlorophyll a and the upper confidence interval”

To: “The association between chlorophyll a and the upper confidence interval”

Figure 2 should first be presented in the Results section.

Change: “This is the case as the probability of exceeding each of the four tested health advisory levels increases as a”

To: “Indeed, the probability of exceeding each of the four tested health advisory levels increased as a”

Change: “We used this association to identify chlorophyll a concentrations that are associated”

To: “We used this association to identify chlorophyll a concentrations that were associated”

I have read this submission. I believe that I have an appropriate level of expertise to confirm that it is of an acceptable scientific standard.

## References

[1] BeaverJManisELoftinKGrahamJPollardAMitchellR: Land use patterns, ecoregion, and microcystin relationships in U.S. lakes and reservoirs: A preliminary evaluation. *Harmful Algae*.2014;36: 10.1016/j.hal.2014.03.005 57-62 10.1016/j.hal.2014.03.005

# Response

**Published:** 2016-06-01

**Authors:** Jeffrey Hollister

**Affiliations:** US EPA, USA

Thank you for your review.  We have just submitted our revisions and expect the new version to be available in the next few days.  Below are our responses to the specific issues you raise.  We feel the paper is stronger after this revision.  Any questions, feel free to contact us or comment here.  Thanks again!

 ## Spatial/Regional Heterogeneity in Chlorophyll/Microcystin realtionship

 We agree that there are likely regional differences and would like to account for this; however, sample sizes for each region vary (67 to 155) and are relatively small.  The resulting conditional probability analysis would have very wide confidence intervals.  Thus, comparison between regions would be difficult and inferring a pattern would not be possible.  We have added additional text in the discussion (second to last paragraph)  that raises this issue.  Additionally, we have added the Beaver et al. reference in this discussion.

 ## Specific Edits

 ### P3

 - Change: “Yuan et al. (2014) explore these associations in detail and control for other related variables. In their analysis they find that total [...]”, To: “Recently, Yuan et al. (2014) explored these associations in detail and controlled for other related variables and found that total [...]”

     - **Response:**  Changed

    

 - Change: “Given these facts, it should be possible to identify chlorophyll a concentrations that would be associated with the [...]”, To:  “These findings suggest that chlorophyll a concentrations could also track the [...]”

     - **Response:** Changed

    

 - Change: “Identifying these associations would provide another tool for [...]”, To: “Identifying this association would provide an important tool for [...]”

     - **Response:** Changed

    

 - Change: “We add to past studies by exploring associations with newly announced advisory [...]”, To: “We build on past studies by exploring associations with the newly announced advisory [...]”

     - **Response:**  Changed

    

 ### P4

 - Change: “Thus, to identify chlorophyll a concentrations of concern we identify the value [...]”, To: “Thus, to identify chlorophyll a concentrations of concern we identified the value [...]”

     - **Response:**  Changed

    

 - Change: “were highly skewed right,”, To: “were highly right skewed,”

     - **Response:** Changed 

    

 - Change: “Lastly, we assess the ability of”, To: “Lastly, we assessed the ability of”

     - **Response:** Changed

    

 - Change: “We use error matrices and calculate total accuracy”, To: “We used error matrices and calculate total accuracy”

     - **Response:** Changed

    

 - Change: “For chlorophyll a, the range was”, To: “Chlorophyll a ranged from”

     - **Response:** Changed 

   

 - Please specify that this chlorophyll a range corresponds to a range from oligotrophic to hypereutrophic lakes.

     - **Response:**  Changed

    

 - Change: “The associations between chlorophyll a and the upper confidence interval”, To: “The association between chlorophyll a and the upper confidence interval”

     - **Response:** Changed 

    

 - Figure 2 should first be (Figure \ref{fig:chla_micro_scatter})presented in the Results section.

     - **Response:** We did not changes this as we fill Figure 2 is not presenting results of our analysis but justifying the approach as is a better fit to be introduced in the Discussion.

     

 - Change: “This is the case as the probability of exceeding each of the four tested health advisory levels increases as a”, To: “Indeed, the probability of exceeding each of the four tested health advisory levels increased as a”

     - **Response:** Changed

     

 - Change: “We used this association to identify chlorophyll a concentrations that are associated”, To: “We used this association to identify chlorophyll a concentrations that were associated” 

     - **Response:** Changed

# Peer review: Referee response for version 1

**DOI:** 10.5256/f1000research.8562.r12688

**Published:** 2016-04-05

**Authors:** Jason W. Marion

**Affiliations:** 1 Department of Environmental Health Science, Eastern Kentucky University, Richmond, KY, USA

**Overview: **The manuscript/article addresses a critical question applicable to recreational and drinking water managers: Can we rapidly predict potentially harmful cyanobacteria blooms using traditional water quality methods? This question is likely to become more relevant according to the most current literature as blooms are expected to increase in frequency in the midst of a warming global climate facing more extreme storm and drought events ^1^^-^^6^. Using the rather large and nationally applicable National Lakes Assessment database for the United States, the authors demonstrate some of the strengths and weaknesses of using chlorophyll *a* as an indicator for at-risk conditions that could warrant management action or follow-up testing for cyanotoxins. The authors are also able to assign action levels or at least share possible action levels for management action using conditional probabilities. The strengths and weaknesses of selected probabilities are described using analyses similar to specificity and sensitivity in the form of accuracy and ‘avoiding type II errors’. The authors do not propose using chlorophyll *a* as a proxy to replace toxin measurement, but as a tool to help facilitate targeted monitoring of toxins during at-risk conditions.

**Overall Comments:** The article is meritorious in that it does provide a meaningful starting point for lakes with no phycocyanin measures and for providing a meaningful starting point for developing some semblance of an action level that could be employed by recreational water and drinking water managers concerned with cyanotoxins. The article does stand to improve significantly in some key areas, which are as follows:

(1) Additional discussion in methods related to the National Lakes Assessment

(2) Additional discussion needed on how the data were organized for data analysis

(3) Improved discussion needed on alternative indicators for cHABs and cyanotoxins not assessed in the NLA

(4) Consideration of region-specific criteria or limitations of national recommendations for chl *a*.

(5) Greater discussion on limitations of NLA and need for model validation/future studies.

(1) Additional discussion needed in methods related to the National Lakes Assessment:

The readership may not be aware of the U.S. NLA performed in 2007. The author(s) should clarify where samples were collected (nearshore or from the surface in the deeper waters). NLA chlorophyll *a *samples were take from the profundal zone rather than the littoral zone. The readership may also be interested in how many chl *a* samples were collected from each lake. Where were the microcystin-LR samples collected?

(2) Additional discussion needed on how the data were organized for data analysis:

Were these samples paired (collected at the same time from the same locale) or are these some type of aggregated value over a lake season? Describing this in the methods will really help for understanding the importance of this work. Paired results (MC-LR and Chl *a* from the same day) are much more impactful for demonstrating the rapid advantage of chl *a* compated to using results that are a seasonal average indicating that the hypereutrophic and eutrophic lakes (ones with the highest chl *a*) are also the ones that are most likely to have a cyanoHAB event sometime during the year.

(3) Improved discussion needed on alternative indicators for cHABs and cyanotoxins not assessed in the NLA: Brief mention is given to phycocyanin (one study), and the additional language (about phycocyanin not always being available for measure and when measured, it is for only measuring pigment and not toxins) is equally relevant for chl *a*. The same *in vivo* handheld fluorometers and continuous monitoring solutions available for chl *a* are now widely available for phycocyanin, often at the same cost as a rapid measure for chl *a*. Phycocyanin, like chl *a, *does not measure toxin either, but phycocyanin in many studies has outperformed chl *a*, and in some studies it has not (especially when toxin concentration is low). Historical records on PC are likely not as great as chlorophyll *a*. Overall, several studies on this topic have been produced in the last two to four years (see Zamyadi and Dorner’s work), with one study using phycocyanin to predict non-alcoholic liver disease presuming a relationship with cyanotoxins (Zhang *et al. *2015)

(4) Consideration desired on region-specific criteria or limitations of national recommendations for chl *a*: With nearly 30% of the lakes in the temperate plains being coded as poor for chlorophyll *a* in the 2007 NLA, what impact would these conditional probabilities have on these lakes? Should the lake managers in this region be monitoring continuously all the time? What are the mean/median chlorophyll *a*  levels for this part of the U.S? Regional variability may be really important and did the conditional probability approach take this into consideration or can it take it into consideration? Is there a way to evaluate if there are significant regional effects in the U.S? For nutrient standards in the U.S. and macroinvertebrate assessments, EPA has had to issue region-specific guidelines/criteria, etc. for some parameters.

(5) Greater discussion needed on limitations of NLA and need for model validation/future studies: The paper fails to address the limitations of the NLA – as a reader, I’m not aware of the limitations. I have much respect for the NLA, but I do have questions regarding the number of samples for each lake. Furthermore, a statement or two discussing the need to validate modeled data may be worthwhile. Is there a way to see if the probabilities actually align with the accuracy and type II error rates predicted by the conditional probability approach?

**Abstract-Specific Comments:** Near the bottom of the abstract, the units seem quite high for microcystins (g/L) rather than micrograms/L. The micro Greek symbol (mu) may have been lost during uploading.

**Results Comments:** (1) In discussing the lake exceedances of the various recommended levels by EPA, the addition of ‘drinking water’ is appropriate in my opinion. Although it is mentioned earlier in the methods, further providing the information in the results is helpful to a novice reader or a person just becoming familiar with drinking water regulations and guidelines, as the U.S. EPA child level may be presumed by a reader to be a level for recreation in a lake rather than a level associated with finished drinking water after water treatment. (2) “All lakes had reported chl *a* concentrations that exceeded detection limits” Does this mean that some were over range? Or  does this mean that “All lakes had detectable levels of chl a”

**Discussion Comments: **The wedge pattern in figure 2 is not apparent in figure 2, however, the logic makes sense and is supported visually by the conditional probability plots in fig 1. If figure 2 could have two lines of best fit (similar to the way some researchers do for funnel plots on publication bias papers), it may be easier to see the wedge shape. 

**Climate Articles Highlighting Current Importance of Topic:**

Harvell, C. D.; Kim, K.; Burkholder, J. M.; Colwell, R. R.; Epstein, P. R.; Grimes, D. J.; Hoffman, E. E.; Lipp,E. K.; Osterhaus, A. D. M. E.; Overstreet, R. M.; Porter, J. W.; Smith, G. W.; Vasta, G. R.Emerging marine diseases – climate links and anthropogenic factors *Science* **2000**, 285, 1505– 1510

Peperzak, L.Climate change and harmful algal blooms in the North Sea *Acta Oecol.* **2003**, 24, 139–144

Edwards, M.; Johns, D. G.; Leterme, S. C.; Svendsen, E.; Richardson, A. J.Regional climate change and harmful algal blooms in the northeast Atlantic *Limnol. Oceanogr.* **2006**, 51 ( 2) 820– 829

Ye, C.; Shen, Z.; Zhang, T.; Feng, M.; Lei, Y.; Zhang, J.Long-term joint effect of nutrients and temperature increase on algal growth in Lake Taihu, China *J* *Environ. Sci.* **2011**, 23 ( 2) 222– 227

Paerl, H. W.; Hall, N. S.; Calandrino, E. S.Controlling harmful cyanobacterial blooms in a world experiencing anthropogenic and climatic-induced change *Sci. Total Environ.* **2011**, 409, 1739– 1745

Davis, T. W.; Berry, D. L.; Boyer, G. L.; Gobler, C. J.The effects of temperature and nutrients on the growth and dynamics of toxic and non-toxic strains of *Microcystis* during cyanobacteria blooms *Harmful Algae* **2009**, 8, 715– 725

**Articles on Phycocyanin and Toxin Indicators Ought to Be Considered:**

Ahn, C.-Y.; Joung, S.-H.; Yoon, S.-K.; Oh, H.-M.Alternative alert system for cyanobacterial bloom, using phycocyanin as a level determinant *J. Microbiol.* **2007**, 45 ( 2) 98– 104.

Makarewicz, J. C.; Boyer, G. L.; Lewis, T. .; Guenther, W.; Atkinson, J.; Arnold, M.Spatial and temporal distribution of the cyanotoxin microcystin-LR in the Lake Ontario ecosystem: Coastal embayments, rivers, nearshore and offshore, and upland lakes *J. Great Lakes Res.* **2009**, 35, 83– 89.

Murby, A. L.Assessing spatial distributions of cyanobacteria and microcystins in N.H. lakes with implications for lake monitoring. Master’s Thesis. University of New Hampshire, **2009**; p 89.

Lehman, E. M.Seasonal occurrence and toxicity of *Microcystis* in impoundments of the Huron River, Michigan, USA *Water Res.* **2007**, 41, 795– 802

Lee C, Marion JW, Cheung M, Lee CS, Lee J. Associations among Human-Associated Fecal Contamination, Microcystis aeruginosa, and Microcystin at Lake Erie Beaches. International journal of environmental research and public health. 2015 Sep 11;12(9):11466-85.

Zamyadi A, Dorner S, Ndong M, Ellis D, Bolduc A, Bastien C, Prévost M. Application of in vivo measurements for the management of cyanobacteria breakthrough into drinking water treatment plants. Environmental Science: Processes & Impacts. 2014;16(2):313-23.

Zhang F, Lee J, Liang S, Shum CK. Cyanobacteria blooms and non-alcoholic liver disease: evidence from a county level ecological study in the United States. Environmental Health. 2015 May 7;14(1):41.

I have read this submission. I believe that I have an appropriate level of expertise to confirm that it is of an acceptable scientific standard, however I have significant reservations, as outlined above.

## References

[1] HarvellCDKimKBurkholderJMColwellRREpsteinPRGrimesDJHofmannEELippEKOsterhausADOverstreetRMPorterJWSmithGWVastaGR: Emerging marine diseases--climate links and anthropogenic factors. *Science*.1999;285(5433) :1505-10 1049853710.1126/science.285.5433.1505

[2] PeperzakL: Climate change and harmful algal blooms in the North Sea. *Acta Oecologica*.2003;24: 10.1016/S1146-609X(03)00009-2 S139-S144 10.1016/S1146-609X(03)00009-2

[3] EdwardsMJohnsDLetermeSSvendsenERichardsonA: Regional climate change and harmful algal blooms in the northeast Atlantic. *Limnology and Oceanography*.2006;51(2) : 10.4319/lo.2006.51.2.0820 820-829 10.4319/lo.2006.51.2.0820

[4] YeCShenZZhangTFanMLeiYZhangJ: Long-term joint effect of nutrients and temperature increase on algal growth in Lake Taihu, China. *J Environ Sci (China)*.2011;23(2) :222-7 2151699510.1016/s1001-0742(10)60396-8

[5] PaerlHWHallNSCalandrinoES: Controlling harmful cyanobacterial blooms in a world experiencing anthropogenic and climatic-induced change. *Sci Total Environ*.2011;409(10) : 10.1016/j.scitotenv.2011.02.001 1739-45 10.1016/j.scitotenv.2011.02.001 21345482

[6] DavisTBerryDBoyerGGoblerC: The effects of temperature and nutrients on the growth and dynamics of toxic and non-toxic strains of Microcystis during cyanobacteria blooms. *Harmful Algae*.2009;8(5) : 10.1016/j.hal.2009.02.004 715-725 10.1016/j.hal.2009.02.004

[7] AhnCYJoungSHYoonSKOhHM: Alternative alert system for cyanobacterial bloom, using phycocyanin as a level determinant. *J Microbiol*.2007;45(2) :98-104 17483793

[8] MakarewiczJBoyerGLewisTGuentherWAtkinsonJArnoldM: Spatial and temporal distribution of the cyanotoxin microcystin-LR in the Lake Ontario ecosystem: Coastal embayments, rivers, nearshore and offshore, and upland lakes. *Journal of Great Lakes Research*.2009;35: 10.1016/j.jglr.2008.11.010 83-89 10.1016/j.jglr.2008.11.010

[9] MurlbyAL: Assessing spatial distributions of cyanobacteria and microcystins in N.H. lakes with implications for lake monitoring.2009;Master's Thesis(University of New Hampshire) :89

[10] LehmanEM: Seasonal occurrence and toxicity of Microcystis in impoundments of the Huron River, Michigan, USA. *Water Res*.2007;41(4) : 10.1016/j.watres.2006.09.030 795-802 10.1016/j.watres.2006.09.030 17208270

[11] LeeCMarionJWCheungMLeeCSLeeJ: Associations among Human-Associated Fecal Contamination, Microcystis aeruginosa, and Microcystin at Lake Erie Beaches. *Int J Environ Res Public Health*.2015;12(9) : 10.3390/ijerph120911466 11466-85 10.3390/ijerph120911466 26378564PMC4586686

[12] ZamyadiADornerSNdongMEllisDBolducABastienCPrévostM: Application of in vivo measurements for the management of cyanobacteria breakthrough into drinking water treatment plants. *Environ Sci Process Impacts*.2014;16(2) : 10.1039/c3em00603d 313-23 10.1039/c3em00603d 24429778

[13] ZhangFLeeJLiangSShumCK: Cyanobacteria blooms and non-alcoholic liver disease: evidence from a county level ecological study in the United States. *Environ Health*.2015;14: 10.1186/s12940-015-0026-7 41 10.1186/s12940-015-0026-7 25948281PMC4428243

# Response

**Published:** 2016-06-01

**Authors:** Jeffrey Hollister

**Affiliations:** US EPA, USA

Thank you for your review.  We have just submitted our revisions and expect the new version to be available in the next few days.  Below are our responses to the specific issues you raise.  We feel the paper is stronger after this revision.  Any questions, feel free to contact us or comment here.  Thanks again!

 ## Additional discussion needed in methods related to the National Lakes Assessment:

 The readership may not be aware of the U.S. NLA performed in 2007. The author(s) should clarify where samples were collected (nearshore or from the surface in the deeper waters). NLA chlorophyll a samples were take from the profundal zone rather than the littoral zone. The readership may also be interested in how many chl a samples were collected from each lake. Where were the microcystin-LR samples collected?

     - **Response:**  We agree that additional information was needed describing the NLA.  We have added this to the first paragraph on the Data section.

 ## Additional discussion needed on how the data were organized for data analysis:

 Were these samples paired (collected at the same time from the same locale) or are these some type of aggregated value over a lake season? Describing this in the methods will really help for understanding the importance of this work. Paired results (MC-LR and Chl a from the same day) are much more impactful for demonstrating the rapid advantage of chl a compated to using results that are a seasonal average indicating that the hypereutrophic and eutrophic lakes (ones with the highest chl a) are also the ones that are most likely to have a cyanoHAB event sometime during the year.

     - **Response:**  We agree and have added some additional wording to the Data section indicating that the samples are taken at the same time.

 ## Improved discussion needed on alternative indicators for cHABs and cyanotoxins not assessed in the NLA: 

 Brief mention is given to phycocyanin (one study), and the additional language (about phycocyanin not always beiThese blooms are expected to increase in frequecy and severity due to the impacts of climate change ng available for measure and when measured, it is for only measuring pigment and not toxins) is equally relevant for chl a. The same in vivo handheld fluorometers and continuous monitoring solutions available for chl a are now widely available for phycocyanin, often at the same cost as a rapid measure for chl a. Phycocyanin, like chl a, does not measure toxin either, but phycocyanin in many studies has outperformed chl a, and in some studies it has not (especially when toxin concentration is low). Historical records on PC are likely not as great as chlorophyll a. Overall, several studies on this topic have been produced in the last two to four years (see Zamyadi and Dorner’s work), with one study using phycocyanin to predict non-alcoholic liver disease presuming a relationship with cyanotoxins (Zhang et al. 2015)

     - **Response:**  We agree that phycocyanin is more closely linked to microcystin than is chl *a*.  Our paragraph mentioning phycocyanin was confusing and did suggest that chl *a* had a stronger association.  Wording of that paragraph has been changed and the Ahn et al paper was added as reference.  We feel that further discussion of phycocyanin, while important, is beyond the scope of our paper with its focus on chlorophyll.

 ## Consideration desired on region-specific criteria or limitations of national recommendations for chl a: 

 With nearly 30% of the lakes in the temperate plains being coded as poor for chlorophyll a in the 2007 NLA, what impact would these conditional probabilities have on these lakes? Should the lake managers in this region be monitoring continuously all the time? What are the mean/median chlorophyll a  levels for this part of the U.S? Regional variability may be really important and did the conditional probability approach take this into consideration or can it take it into consideration? Is there a way to evaluate if there are significant regional effects in the U.S? For nutrient standards in the U.S. and macroinvertebrate assessments, EPA has had to issue region-specific guidelines/criteria, etc. for some parameters.

     - **Response:**  We agree that there are likely regional differences and would like to account for this; however, sample sizes for each region vary (67 to 155) and are relatively small.  The resulting conditional probability analysis would have very wide confidence intervals.  Thus, comparison between regions would be difficult and inferring a pattern would not be possible.  We have added additional text in the discussion (second to last paragraph)  that raises this issue.  Additionally, we have added the Beaver et al. reference in this discussion.

 ## Greater discussion needed on limitations of NLA and need for model validation/future studies: 

 The paper fails to address the limitations of the NLA – as a reader, I’m not aware of the limitations. I have much respect for the NLA, but I do have questions regarding the number of samples for each lake. Furthermore, a statement or two discussing the need to validate modeled data may be worthwhile. Is there a way to see if the probabilities actually align with the accuracy and type II error rates predicted by the conditional probability approach?

     - **Response:**  We added a paragraph to the discussions about validation and the single sample limitations of the NLA.

 ## Specific Edits

 ### Abstract-Specific Comments: 

 - Near the bottom of the abstract, the units seem quite high for microcystins (g/L) rather than micrograms/L. The micro Greek symbol (mu) may have been lost during uploading.

     - **Response:** Oops!  Looks like it did occur during upload.  Have double checked the final for the proper units.

   

 ### Results Comments: 

 - In discussing the lake exceedances of the various recommended levels by EPA, the addition of ‘drinking water’ is appropriate in my opinion. Although it is mentioned earlier in the methods, further providing the information in the results is helpful to a novice reader or a person just becoming familiar with drinking water regulations and guidelines, as the U.S. EPA child level may be presumed by a reader to be a level for recreation in a lake rather than a level associated with finished drinking water after water treatment. 

     - **Response:**  Added this in.

 - “All lakes had reported chl a concentrations that exceeded detection limits” Does this mean that some were over range? Or  does this mean that “All lakes had detectable levels of chl a”

     - **Response:** Changed wording to: All lakes had detectable levels of ... 

   

 ### Discussion Comments: 

 - The wedge pattern in figure 2 is not apparent in figure 2, however, the logic makes sense and is supported visually by the conditional probability plots in fig 1. If figure 2 could have two lines of best fit (similar to the way some researchers do for funnel plots on publication bias papers), it may be easier to see the wedge shape. 

     - **Response:**  Agreed that the wedge is not pronounced.  We have changed the wording to better describe the pattern.  Also, we disagree with the lines of best fit as those would then imply some sort of linear (presumably) pattern that we are not actually highlighting.  Additionally the added lines would then require discussion and would detract from the focus on conditional probability.

# Peer review: Referee response for version 1

**DOI:** 10.5256/f1000research.8562.r12330

**Published:** 2016-02-22

**Authors:** Alan E. Wilson

**Affiliations:** 1 School of Fisheries, Aquaculture, and Aquatic Sciences, College of Agriculture, Auburn University, Auburn, AL, USA

**Title and Abstract:**

For clarity, the authors might consider replacing “various” with “World Health Organization and U.S. Environmental Protection Agency”.  

**Article content:**

Using publicly available data produced from the 2007 USEPA National Lakes Assessment, the authors use conditional probability analysis to develop four chlorophyll concentration thresholds associated with two USEPA microcystin drinking water advisory targets, one WHO microcystin drinking water advisory target, and one WHO microcystin recreational use target.  For most water resource managers, chlorophyll is much easier and cheaper to measure than microcystin. Given the threat that microcystins pose to human health, the purpose of this study is valuable. With that said, this study significantly overlaps with Yuan *et al. *(2014).  The current study adds new advisory microcystin targets recently established by USEPA and uses a different statistical approach than Yuan *et al. *2014. The authors cite relatively few studies. I think the authors need to more broadly consider the existing literature and describe how their findings relate to and build from past studies. Below, I provide some related studies that the authors might want to consider. I am certain that I have missed other relevant studies. 

Ahn *et al. *(2011)

Beaver *et al. *(2014)

Chan *et al. *(2007)

Conti *et al. *(2005)

Dolman *et al. *(2012)

Downing *et al. *(2000)

Giani *et al. *(2005)

Graham *et al. *(2004)

Graham *et al. *(2010)

Jacoby *et al. *(2015)

Kotak *et al. *(2000)

Marion *et al. *(2012)

Orihel *et al. *(2012)

Sarnelle *et al. *(2010)

Scott and Haggard (2015)

Sinang *et al. *(2015)

Stow *et al. *(2015)

Su *et al. *(2015)

Yuan and Pollard *et al. *(2015)

Based on the 2007 National Lakes Assessment report, roughly two-thirds of the waterbodies reported no detectable microcystin (detection limit = 0.05 ug/L) despite covering a huge range of chlorophyll concentrations.  And, Fig 2 suggests that a large number of sites had barely detectable concentration of microcystin across a wide range of chlorophyll.  It is not clear from the text how the authors dealt with waterbodies with undetectable or barely detectable microcystin concentrations.  

Presenting histograms of chlorophyll and microcystin concentrations for the study lakes would be useful. 

I am not an expert on conditional probability analysis. Based on the authors’ text (second paragraph in Analytical Methods section), it appears that this analysis considers multiple events over time. If their dataset includes single measurements in a waterbody, I don’t understand where the temporal component comes into the analysis. Again, I could be totally misunderstanding how this analysis works and should probably read the relevant references the authors provided. 

Based on increasing error in the conditional probability plots as chlorophyll increases, the reported chlorophyll thresholds should not include significant digits (i.e., ± 0.1) but instead be whole numbers.

I would organize the information in table 1 by either concentration (low to high) or advisory type (drinking or recreational) and concentration (low to high). It might also be useful to include the number of lakes represented in each category based on microcystin. In table 2, I would add the specific microcystin concentration target under each advisory type to avoid having to look back at table 1 for these data.

**Conclusions:**

The purpose of this study is to use a simple measurement (chlorophyll) to determine the threat that microcystins pose to a waterbody relative to existing microcystin concentration targets. Most waterbodies lacked microcystin and Figure 2 clearly shows that there are a huge number of waterbodies across a large chlorophyll range that apparently had microcystin concentrations at the detection limit of 0.05 ug/L. I am concerned about the microcystin data at the detection limit. They appear to be false positives. I agree with the authors who acknowledged that high chlorophyll is not always a good predictor of high microcystin. What should be done for those waterbodies with high concentrations of chlorophyll but that had no or barely detectable microcystin?

**Data:**

I am confused about the data collected and available for the 2007 National Lakes Assessment. For example, I organized this dataset in July 2010 and found that 1158 lakes were sampled once (1152 of these lakes included data for both chlorophyll and microcystin) and 95 of the 1158 originally sampled lakes were sampled a second time in 2007. Yuan et al. 2014 (Freshwater Biology) used data for 1077 sampled lakes. The current study (as well as the National Lakes Assessment website and report) describes data for 1028 lakes. Clarity about these discrepancies is not necessarily the authors’ job, but it would be good to understand why the differences exist across these datasets. Also, for this study, how were data used for lakes sampled twice in 2007?  

Although all of the National Lakes Assessment data are publicly available, the authors should provide the dataset that they used for this study.

I have read this submission. I believe that I have an appropriate level of expertise to confirm that it is of an acceptable scientific standard, however I have significant reservations, as outlined above.

## References

[1] AhnCOhHParkY: EVALUATION OF ENVIRONMENTAL FACTORS ON CYANOBACTERIAL BLOOM IN EUTROPHIC RESERVOIR USING ARTIFICIAL NEURAL NETWORKS1. *Journal of Phycology*.2011;47(3) : 10.1111/j.1529-8817.2011.00990.x 495-504 10.1111/j.1529-8817.2011.00990.x 27021978

[2] BeaverJManisELoftinKGrahamJPollardAMitchellR: Land use patterns, ecoregion, and microcystin relationships in U.S. lakes and reservoirs: A preliminary evaluation. *Harmful Algae*.2014;36: 10.1016/j.hal.2014.03.005 57-62 10.1016/j.hal.2014.03.005

[3] ChanWSRecknagelFCaoHParkHD: Elucidation and short-term forecasting of microcystin concentrations in Lake Suwa (Japan) by means of artificial neural networks and evolutionary algorithms. *Water Res*.2007;41(10) : 10.1016/j.watres.2007.02.001 2247-55 10.1016/j.watres.2007.02.001 17408719

[4] ContiALGuerreroJMRegueiraJM: Levels of microcystins in two Argentinean reservoirs used for water supply and recreation: differences in the implementation of safe levels. *Environ Toxicol*.2005;20(3) : 10.1002/tox.20107 263-9 10.1002/tox.20107 15892071

[5] DolmanAMRückerJPickFRFastnerJRohrlackTMischkeUWiednerC: Cyanobacteria and cyanotoxins: the influence of nitrogen versus phosphorus. *PLoS One*.2012;7(6) : 10.1371/journal.pone.0038757 e38757 10.1371/journal.pone.0038757 22719937PMC3376147

[6] DowningJWatsonSMcCauleyE: Predicting Cyanobacteria dominance in lakes. *Canadian Journal of Fisheries and Aquatic Sciences*.2001;58(10) : 10.1139/cjfas-58-10-1905 1905-1908 10.1139/cjfas-58-10-1905

[7] GianiABirdDPrairieYLawrenceJ: Empirical study of cyanobacterial toxicity along a trophic gradient of lakes. *Canadian Journal of Fisheries and Aquatic Sciences*.2005;62(9) : 10.1139/f05-124 2100-2109 10.1139/f05-124

[8] GrahamJLJonesJRJonesSBDowningJAClevengerTE: Environmental factors influencing microcystin distribution and concentration in the Midwestern United States. *Water Res*.2004;38(20) : 10.1016/j.watres.2004.08.004 4395-404 10.1016/j.watres.2004.08.004 15556214

[9] GrahamJLLoftinKAMeyerMTZieglerAC: Cyanotoxin mixtures and taste-and-odor compounds in cyanobacterial blooms from the Midwestern United States. *Environ Sci Technol*.2010;44(19) : 10.1021/es1008938 7361-8 10.1021/es1008938 20831209

[10] JacobyJBurghdoffMWilliamsGReadLHardyJ: Dominant factors associated with microcystins in nine midlatitude, maritime lakes. *Inland Waters*.2015;5(2) : 10.5268/IW-5.2.808 187-202 10.5268/IW-5.2.808

[11] KotakBLamAPrepasEHrudeyS: Role of chemical and physical variables in regulating microcystin-LR concentration in phytoplankton of eutrophic lakes. *Canadian Journal of Fisheries and Aquatic Sciences*.2000;57(8) : 10.1139/f00-091 1584-1593 10.1139/f00-091

[12] MarionJWLeeJWilkinsJRLemeshowSLeeCWaletzkoEJBuckleyTJ: In vivo phycocyanin flourometry as a potential rapid screening tool for predicting elevated microcystin concentrations at eutrophic lakes. *Environ Sci Technol*.2012;46(8) : 10.1021/es203962u 4523-31 10.1021/es203962u 22404495

[13] OrihelDBirdDBrylinskyMChenHDonaldDHuangDGianiAKinniburghDKlingHKotakBLeavittPNielsenCReedykSRooneyRWatsonSZurawellRVinebrookeRSmithR: High microcystin concentrations occur only at low nitrogen-to-phosphorus ratios in nutrient-rich Canadian lakes. *Canadian Journal of Fisheries and Aquatic Sciences*.2012;69(9) : 10.1139/f2012-088 1457-1462 10.1139/f2012-088

[14] SarnelleOMorrisonJKaulRHorstGWandellHBednarzR: Citizen monitoring: Testing hypotheses about the interactive influences of eutrophication and mussel invasion on a cyanobacterial toxin in lakes. *Water Res*.2010;44(1) : 10.1016/j.watres.2009.09.014 141-50 10.1016/j.watres.2009.09.014 19781732

[15] ScottJTHaggardBE: Implementing Effects-Based Water Quality Criteria for Eutrophication in Beaver Lake, Arkansas: Linking Standard Development and Assessment Methodology. *J Environ Qual*.2015;44(5) : 10.2134/jeq2015.01.0020 1503-12 10.2134/jeq2015.01.0020 26436267

[16] SinangSReichwaldtEGhadouaniA: Local nutrient regimes determine site-specific environmental triggers of cyanobacterial and microcystin variability in urban lakes. *Hydrology and Earth System Sciences*.2015;19(5) : 10.5194/hess-19-2179-2015 2179-2195 10.5194/hess-19-2179-2015

[17] StowCAChaYJohnsonLTConfesorRRichardsRP: Long-term and seasonal trend decomposition of Maumee River nutrient inputs to western Lake Erie. *Environ Sci Technol*.2015;49(6) : 10.1021/es5062648 3392-400 10.1021/es5062648 25679045

[18] SuXXueQSteinmanADZhaoYXieL: Spatiotemporal Dynamics of Microcystin Variants and Relationships with Environmental Parameters in Lake Taihu, China. *Toxins (Basel)*.2015;7(8) : 10.3390/toxins7083224 3224-44 10.3390/toxins7083224 26295260PMC4549747

[19] YuanLPollardA: Deriving nutrient targets to prevent excessive cyanobacterial densities in U.S. lakes and reservoirs. *Freshwater Biology*.2015;60(9) : 10.1111/fwb.12620 1901-1916 10.1111/fwb.12620

[20] YuanLPollardAPatherSOliverJD'AngladaL: Managing microcystin: identifying national-scale thresholds for total nitrogen and chlorophylla. *Freshwater Biology*.2014;59(9) : 10.1111/fwb.12400 1970-1981 10.1111/fwb.12400

# Response

**Published:** 2016-06-01

**Authors:** Jeffrey Hollister

**Affiliations:** US EPA, USA

## I think the authors need to more broadly consider the existing literature and describe how their findings relate to and build from past studies. Below, I provide some related studies that the authors might want to consider. 

     - **Response:**  First thanks for the fantastic list of refs!  Having it linked with this publication is a resource in and of itself.  We have looked at those carefully and have added several: including Ahn et al, Beaver et al, Yuan and Pollard, and Marion et al.  We have not added signficantly to the background on this paper because our goal was to keep this research communication short and focused on on the chl and microcystin relationship.   

 ## Based on the 2007 National Lakes Assessment report, roughly two-thirds of the waterbodies reported no detectable microcystin (detection limit = 0.05 ug/L) despite covering a huge range of chlorophyll concentrations.  And, Fig 2 suggests that a large number of sites had barely detectable concentration of microcystin across a wide range of chlorophyll.  It is not clear from the text how the authors dealt with waterbodies with undetectable or barely detectable microcystin concentrations.

     - **Response:**  We have added some text to the Data section indicating how we deal with the detection limit.  We feel it is important to keep these values in the analysis as removing them would inflate our confidence around the conditional probabilities.  We hope this is clearer in our revision.

 ## Presenting histograms of chlorophyll and microcystin concentrations for the study lakes would be useful. 

     - **Response:**  We have chosen to present the distribution information in text and present for both chlorophyll and microcystin the range, mean, and median.  Figure 2 also indicates the distribution of both.  Lastly, the data are availble via [code from the GitHub repository](https://github.com/USEPA/Microcystinchla/blob/master/R/get_nla.R).

 ## I am not an expert on conditional probability analysis. Based on the authors’ text (second paragraph in Analytical Methods section), it appears that this analysis considers multiple events over time. If their dataset includes single measurements in a waterbody, I don’t understand where the temporal component comes into the analysis. Again, I could be totally misunderstanding how this analysis works and should probably read the relevant references the authors provided. 

     - **Response:**  We have added some additional text in the methods about the NLA as well as in the Discussion on NLA limitations.  In short, this is not a temporal analysis and is based on a single snap shot.

 ## Based on increasing error in the conditional probability plots as chlorophyll increases, the reported chlorophyll thresholds should not include significant digits (i.e., ± 0.1) but instead be whole numbers.

     - **Response:**  Done. NEED TO DO on table already in overleaf

 ## I would organize the information in table 1 by either concentration (low to high) or advisory type (drinking or recreational) and concentration (low to high). It might also be useful to include the number of lakes represented in each category based on microcystin. 

     - **Response:** Table  re-orderd based on concentration.  Number of lakes (as percentage) included in text.  Need to do directly on table in overleaf.

 ## In table 2, I would add the specific microcystin concentration target under each advisory type to avoid having to look back at table 1 for these data.

     - **Response:**  Done.  Need to transfer to overleaf.

 ## Most waterbodies lacked microcystin and Figure 2 clearly shows that there are a huge number of waterbodies across a large chlorophyll range that apparently had microcystin concentrations at the detection limit of 0.05 ug/L. I am concerned about the microcystin data at the detection limit. They appear to be false positives. I agree with the authors who acknowledged that high chlorophyll is not always a good predictor of high microcystin. What should be done for those waterbodies with high concentrations of chlorophyll but that had no or barely detectable microcystin?

     - **Response:** We added some discussion about this in the last paragraph of the Data section.  We feel that these should be left in as removing them would erroneously inflate our confidence intervals and impact the conditional probabilities.  Essentially these are lakes with very low microcystin but widely varying chlorophyll values.   

 ## I am confused about the data collected and available for the 2007 National Lakes Assessment. For example, I organized this dataset in July 2010 and found that 1158 lakes were sampled once (1152 of these lakes included data for both chlorophyll and microcystin) and 95 of the 1158 originally sampled lakes were sampled a second time in 2007. Yuan et al. 2014 (Freshwater Biology) used data for 1077 sampled lakes. The current study (as well as the National Lakes Assessment website and report) describes data for 1028 lakes. Clarity about these discrepancies is not necessarily the authors’ job, but it would be good to understand why the differences exist across these datasets. Also, for this study, how were data used for lakes sampled twice in 2007?  

     - **Response:** We share your confusion!  There are many "types" of samples included with the raw NLA data.  For this analysis, we only used the probability samples (i.e. no reference samples) and only used the first visit to a lake.  Additionally, lakes that had no data reported for either chl or microsystin were not included.  As noted, this results in 1028 samples

 ## Although all of the National Lakes Assessment data are publicly available, the authors should provide the dataset that they used for this study. 

     - **Response:** Code to access the data is available from [USEPA/microcysinchla](https://github.com/USEPA/microcystinchla).  We have also added in a static .csv file to this repository of the data used for our analysis. This is listed in the "Data and software availability" section.

 Thank you for your review.  We have just submitted our revisions and expect the new version to be available in the next few days.  Below are our responses to the specific issues you raise.  We feel the paper is stronger after this revision.  Any questions, feel free to contact us or comment here.  Thanks again!

 ## Specific Edits:

 ### Title and Abstract: 

 - For clarity, the authors might consider replacing “various” with “World Health Organization and U.S. Environmental Protection Agency”. 

     - **Response:**  As this has already been indexed, we thought it best to limit the edits to the title.
